# Differential Profile of Ultrasound Findings Associated with Malignancy in Mixed and Solid Thyroid Nodules in an Elderly Female Population

**DOI:** 10.1155/2014/761653

**Published:** 2014-06-23

**Authors:** María Inés Vera, Tomás Meroño, María Agustina Urrutia, Carina Parisi, Yanina Morosan, Melanie Rosmarin, Marta Schnitman, Fernando Brites, Silvio Grisendi, María Sol Serrano, Wilfredo Luciani, Leonardo Serrano, Carlos Zuk, Guillermo De Barrio, Claudia Cejas, María Cristina Faingold, Gabriela Brenta

**Affiliations:** ^1^Department of Endocrinology and Metabolism, Dr. Cesar Milstein Hospital, 951 La Rioja Street, C1221ACI Buenos Aires, Argentina; ^2^School of Pharmacy and Biochemistry, UBA, 954 Junín Street, C1113AAD Buenos Aires, Argentina; ^3^Department of Cyto-Pathology, Dr. Cesar Milstein Hospital, 951 La Rioja Street, C1221ACI Buenos Aires, Argentina; ^4^Department of Surgery, Dr. Cesar Milstein Hospital, 951 La Rioja Street, C1221ACI Buenos Aires, Argentina; ^5^Department of Medical Imaging, Dr. Cesar Milstein Hospital, 951 La Rioja Street, C1221ACI Buenos Aires, Argentina

## Abstract

*Objective.* Ultrasonographic characteristics are associated with thyroid malignancy. Our aim was to compare the diagnostic value of ultrasound features in the detection of thyroid malignancy in both solid and mixed nodules. *Methods.* We prospectively studied female patients (≥50 years) referred to ultrasound-guided fine needle aspiration biopsy. Ultrasound features considered suspicious were hypoechogenicity, microcalcifications, irregular margins, high anteroposterior (AP)/axial-ratio, and absent halo. Associations were separately assessed in mixed and solid nodules. *Results.* In a group of 504 elderly female patients (age = 69 ± 8 years), the frequency of malignant cytology was 6%. Thirty-one percent of nodules were mixed and 60% were solid. The rate of malignant cytology was similar for mixed and solid nodules (7.4 versus 5.8%, *P*: 0.56). While in mixed nodules none of the ultrasound characteristics were associated with malignant cytology, in solid nodules irregular margins and microcalcifications were significant (all *P* < 0.05). The combination of irregular margins and/or microcalcifications significantly increased the association with malignant cytology only in solid nodules (OR: 2.76 (95% CI: 1.25–6.10), *P*: 0.012). *Conclusions.* Ultrasound features were of poor diagnostic value in mixed nodules, which harbored malignant lesions as often as solid nodules. Our findings challenge the recommended minimal size for ultrasound-guided fine needle aspiration biopsy in mixed nodules.

## 1. Introduction

Thyroid nodules are among the most frequent disorders referred to endocrinological practice. While 4–7% of the general adult population has palpable thyroid nodules, more than 50% has thyroid nodules on ultrasonography (US) [1]. Furthermore, an increasing incidence of thyroid cancer has been reported worldwide, and a possible explanation is the more frequent practice of imaging studies. Regarding gender, thyroid nodules, as well as thyroid cancer, are more common in women than in men [2, 3]. In fact, out of 5234 subjects aged >60 years in Framingham, clinically apparent thyroid nodules were present in 6.4% of women and 1.5% of men [2]. Furthermore, ageing is also associated with an increased prevalence of nodular goiter. Moreover, it has been shown that while nodular goiter is present in 2.7 and 2.0% of young women and men, respectively, the mean prevalence in women and men over 55 years is 18.0 and 14.5%, respectively [4]. More importantly, thyroid cancer in elderly patients portends a more aggressive behavior [5] and mortality rates are higher in patients over 45 years old at diagnosis [6]. Considering that life expectancy has increased, diagnostic procedures in the aged population should improve the detection of thyroid cancer and help to avoid unnecessary surgical procedures.

US is the most common diagnostic tool used before fine needle aspiration biopsy (FNAB) in thyroid nodular disease. Certain US features, such as solid echostructure, hypoechogenicity, irregular margins, absence of halo, microcalcifications, anteroposterior (AP) diameter larger than axial diameter (taller than wider shape), and intranodular vascularization, have all been proposed to help select nodules that have to be biopsied [7, 8]. However, the diagnostic impact of these US features has not been fully explored in the elderly population.

Mixed nodules represent 15–54% of all surgically excised thyroid nodules [9–12] and although the proportion of malignancy among mixed nodules has been reported to be between 2% and 18% [12], in general, their relevance is minimized because they are believed to represent a degenerative process arising from benign lesions. In fact, the American thyroid association (ATA) Guidelines [8] state that while all solid nodules larger than 1 cm should undergo FNAB, mixed cystic-solid nodules should be biopsied only when size is larger than 1.5 or 2.0 cm in the presence of suspicious US findings, or when size is above 2.0 cm if no suspicious US findings are present.

Few studies analyzed US features associated with thyroid cancer taking into consideration age, gender, and in particular the distinction between mixed and solid nodules. Our aim was to compare the diagnostic value of US features for the detection of thyroid malignancy in solid and mixed nodules in an elderly female population.

## 2. Materials and Methods

### 2.1. Study Cohort and Protocol

Our study was performed at the Dr. Cesar Milstein Hospital, which receives all the patients belonging to the National Institute of Social Services for Retirees and Pensioners (INSSJP), referred for US-guided FNAB in the city of Buenos Aires. The population selected for this study consisted of 504 female patients (age = 69 ± 8 years) referred to the Dr. Cesar Milstein Hospital between June 2008 and May 2012 for FNAB. All patients live in Buenos Aires, which is considered an iodine-rich region in Argentina. The main reasons for referral were a neck mass that was visible and/or palpable or that had been found incidentally in a previous CT or US. The study was approved by the ethical committee of our institution and all patients signed an informed consent form. Clinical and US details were prospectively collected in a special form that was filled out right before FNAB was performed. The US characteristics of the nodules were described by the operator to a technician who was in charge of writing down the information provided.

### 2.2. Image Analysis

US characteristics were assessed by real-time US in each thyroid nodule biopsied. These included type of echostructure, echogenic pattern (the nodule echogenicity was compared to normal thyroid and strap muscles), shape of margins, presence of halo (complete or partial) and microcalcifications, and the 3 diameters of the nodule in mm. Microcalcifications were defined as tiny, punctate hyperechoic foci, without comet-tail artifacts that may represent precipitated colloid material. Coarse calcifications were not considered for the analysis. In Table 1 are depicted the US characteristics and each of their possible variants that were described in each nodule.

Mixed or partially cystic nodules were defined by the presence of an anechoic component. If a nodule had at least 25% of anechoic component it was considered mixed. Completely anechoic nodules were excluded from the study. US features in mixed nodules were evaluated based on their solid component.

### 2.3. US-Guided Fine Needle Aspiration Procedure

A Mindray DC-3 (Shenzhen, China) Doppler-echo machine and a 7.5–10 MHz linear-array probe were used to guide all FNABs in real time. Biopsies were performed using a 23-gauge needle, and visualization of the tip of the needle inside the nodule helped to monitor the correct site for biopsy. To select the nodule for biopsy, we followed the ATA guidelines [8]. US-guided FNABs were carried out by one of the three operators of our institution, each having more than 15 years of experience with this procedure. In some patients in whom it was difficult to place the probe due to the shape of the neck, the skin-marking technique was employed to perform FNAB [13]. At least 2–6 needle passages were performed in each nodule. Material obtained from FNABs was smeared on glass slides, which were immediately placed in 95% alcohol for Papanicolau staining and sent to the Pathology Department.

### 2.4. Cytological Analysis

Cytological analysis was performed independently by two pathologists. Validation of this procedure by cytohistological correlation in our institution was previously reported [13]. In the present series, 46 subjects were referred to surgery and cancer was confirmed in 20 of them (43%). After excluding indeterminate cases, cytology showed a significant concordance with histological results (kappa coefficient = 0.68, *P* < 0.001). For this study we started recruiting patients before the Bethesda system was introduced in our hospital practice. Therefore, we continued using our former classification. Accordingly, results were classified as benign, insufficient, indeterminate, and malignant [13]. Results suspicious of papillary thyroid cancer were included in the malignant category. However, those suspicious of follicular/Hurthle cell neoplasm were included in the indeterminate category together with results consistent with atypical cells of undetermined significance.

### 2.5. Statistical Analysis

For statistical analysis, one nodule was considered per patient. In patients who had two nodules biopsied, with both showing benign cytology, the nodule with the largest longitudinal diameter was considered for analysis. In no case did both nodules biopsied show malignant cytology. For statistical analysis, malignant cytology was considered as the gold standard. US features were considered as dichotomous variables defined by the presence or absence of the US characteristics. Taller than wider shape was defined as a value of AP/axial diameters >1. When comparing the prevalence of the different echographic characteristics between mixed and solid nodules, the Chi-square test was employed. After discarding indeterminate and insufficient cytology results, a subpopulation of 391 nodules with benign and malignant cytological results was obtained. The associations among the different US characteristics and malignant cytology were analyzed with the Chi-square test. Likelihood ratios (LR) and posttest probabilities were calculated from 2 × 2 tables [14]. Pretest probability was defined as 0.06 (estimated from the whole sample). Data distribution was assessed by the Shapiro-Wilks test. Continuous variables were compared using the Student's *t*-test or Mann-Whitney *U* test according to data distribution. Multiple logistic regression analysis was employed to find the combination of US characteristics best associated with the presence of malignant cytology. Normally distributed variables are presented as mean ± S.D. and skewed variables as median (interquartile range). A *P* value < 0.05 was considered significant. All analyses were performed with SPSS 17.0 statistical software (IBM, Chicago, IL, USA).

## 3. Results

Attesting to the elderly female population included (*n* = 504), the age distribution of the patients was 6%: 50–60 years; 53%: 60–70 years; 34%: 70–80 years; and 7%: > 80 years (mean age = 69 ± 8 years). Among the overall population of nodules, the following cytological results were obtained: 6% insufficient, 8% indeterminate, 80% benign, and 6% malignant. Cytological results were similar across age intervals (data not shown).

According to their echostructure, nodules were classified as mixed (31%), solid (60%), cystic (2%), and spongiform (1%), while in 6% of cases echostructure data was missing. The prevalence of other suspicious US features among all nodules was hypoechoicity (54%), irregular margins (38%), microcalcifications (39%), absence of halo (31%), and taller than wider shape (36%). Subsequently, only mixed and solid nodules were analyzed (*n* = 462). General characteristics of these 2 groups are depicted in Table 2. Mixed nodules presented larger diameters, in accordance with the indications stated in the ATA guidelines for FNAB [8]. Taller than wider shape was the only echographic feature in which a lower prevalence was observed in mixed nodules, though this result was not statistically significant. Noteworthy, there was no difference in the prevalence of malignant cytology between mixed and solid nodules. Moreover, the number of cases in which cancer (80% papillary carcinoma) was confirmed by histology was similar for both types of nodules (5.7 versus 3.7%, *P* = 0.85).

The associations between US features and malignant cytology in mixed and solid nodules are shown in Table 3. Strikingly, for mixed nodules, none of the US characteristics analyzed were significantly associated with malignant cytology (Table 3). Nonetheless, the positive LR was significantly increased for mixed nodules with no halo. By contrast, in solid nodules, irregular margins and microcalcifications were significantly associated with malignant cytology and increased the positive LR.

Despite the lack of significant association with malignant cytology, it is worth noting that US features that improved the posttest probability for malignant cytology in mixed nodules were absence of halo and taller than wider shape. By contrast, in solid nodules, irregular margins and microcalcifications were characteristics that significantly improved the posttest probability of malignant cytology (Table 3). Interestingly, taller than wider shape slightly improved the posttest probability in both types of nodules. Although mixed nodules were larger than solid ones, among malignant nodules there was no difference in any of the three measured diameters (longitudinal: 2.0 (1.7–2.7) versus 1.8 (1.3–2.4) cm; AP: 2.0 (1.0–2.1) versus 1.7 (0.9–2.0) cm; and axial: 2.0 (1.0–3.0) versus 1.2 (0.9–1.7) cm, all *P* > 0.05 for mixed and solid nodules, resp.).

Given that microcalcifications and irregular margins were the 2 parameters best associated with malignant cytology in solid nodules, we ran a multiple logistic regression analysis to evaluate if a combination of both US features was associated with malignant cytology. For mixed nodules, neither age nor the compound between irregular margins and microcalcifications was significantly associated with malignant cytology. As for solid nodules, the presence of irregular margins/microcalcifications was statistically significant, thus showing that the odds for malignant cytology gradually increase 2.7-fold if a nodule presents one or both US features (Table 4).

## 4. Discussion

The most important finding of this study was the identification of different profiles of US characteristics associated with malignant cytology, exhibited by mixed and solid thyroid nodules. For mixed nodules, the association of absence of halo and malignant cytology showed a trend, while for solid nodules the association of irregular margins and microcalcifications with malignant cytology proved significant. Overall, these results suggest that in an aged population (94% > 60 years), US characteristics perform quite acceptably to identify suspicious solid nodules. However, this is not true for mixed nodules, in which US suspicious characteristics were not efficient to guide FNAB. Further concerns are raised by the facts that (a) malignancy was found to a similar extent in mixed and solid nodules and (b) similar diameters were observed in malignant mixed and solid thyroid nodules. Based on these findings, mixed nodules should be biopsied at the same size thresholds as solid nodules, as opposed to what the current ATA guidelines suggest.

US is a helpful tool in the detection of thyroid cancer, and several scores have been recently proposed to select the nodule which should be biopsied [15, 16]. Papini et al. reported that the combination of solid echostructure with hypoechogenic pattern has 87% sensitivity for thyroid cancer, albeit with low specificity and low positive predictive value [17]. In addition, microcalcifications have also been extensively studied and described as one of the main predictors of papillary thyroid cancer [18]. Consistent with these findings, in the present study microcalcifications were significantly associated with malignant cytology, but only for solid nodules. Irregular margins are another US feature which has proved to be highly specific for thyroid malignancy [17]. In fact, in solid nodules we observed that irregular margins were associated with malignant cytology, above any other US feature. A possible explanation for such observation is that elderly patients might have had thyroid cancer long enough to enable the occurrence of local invasion.

As to the taller than wider shape, this feature exhibited high specificity for malignancy in both mixed and solid nodules. The shape of the “taller than wider” nodule, although not extensively described, has been previously mentioned in the literature where, in agreement with our work, it was found to be specific but with reduced sensitivity [19]. In breast cancer, this concept of AP growth is widely accepted [20] but is less so for the thyroid. US reports of thyroid nodules often describe only one diameter, in particular, the longitudinal diameter and lack information regarding the AP or axial diameters. This neglected information could be, however, very helpful to understand tumor growth. Malignant tumors have a centrifugal growth pattern while benign nodules tend to expand in the longitudinal axis of the thyroid lobe, in parallel with the skin [19].

Few studies have specifically addressed US features in mixed thyroid nodules [21]. In contrast with our results, Sohn et al. [22] described that US features in mixed nodules were significant predictors of thyroid cancer. In their study, the rate of malignancy was 4% in a series of 316 mixed nodules. Moreover, 31.6% of thyroid nodules with suspicious US features were malignant compared to only 2.7% of those without them. Their findings led to the suggestion that mixed nodules without suspicious US features could be followed by US even if they grew in size. However, among the 316 mixed nodules, the proportion of nodules with suspicious US features was really low, only 6%. Additionally, in some US-based classifications to detect malignancy in mixed nodules, some of the US features considered are different from those generally described [21, 23]. These include eccentric configuration with an acute angle, macrolobulated or irregular free margin of the solid component, centripetal vascularity in the pedicle, and associated cervical lymphadenopathy with intranodal cystic components or microcalcifications [21, 24, 25]. Based on these features, Kim et al. [26] have proposed a US-based classification system to differentiate benign from malignant in partially cystic thyroid nodules. They proved the diagnostic accuracy of US for thyroid nodules to be very high in a large series of 1289 nodules, among which 234 were partially cystic. However, in line with our results, they used different classification criteria for solid and mixed nodules. In this context, the use of a specific classification only for mixed nodules is highly recommended. Future studies regarding the accuracy of US features to guide FNAB in mixed thyroid nodules are warranted.

Another consideration is that it would be expected that as nodules exhibit more US suspicious features, the odds of presenting malignant cytology should markedly increase. In fact, Kwak et al. [27] showed that the risk of malignancy increased in parallel with the number of suspicious US features. However, in the present study this was only true for solid nodules in which irregular margins and/or microcalcifications increased the odds for malignant cytology 2.7-fold. Further studies are needed in order to reach consensus about the US characteristics that should be considered for FNAB.

Although most of the previous studies reported the sensitivity and specificity of different US characteristics to detect malignant nodules, calculation of the LR and posttest probability highlights some clinical aspects of our results. In this regard, though sensitivity and specificity are crucial to describe the performance of a test, their interpretation in clinical practice is not as simple as positive LR and posttest probabilities. The LR has an advantage over sensitivity and specificity because it is less likely to change with the prevalence of the disorder. Furthermore, the posttest probability shows how the presence of a certain US feature improves the chance to find malignant cytology in a particular nodule.

We acknowledge our limitation regarding cytological instead of pathological criteria for malignancy. However, this was decided given the high cytopathological correlation found in our Institution. Also, information regarding vascularity was not included in the analyses due to a high prevalence of missing data. The selection of a population well-defined by sex and age (94% > 60 years) represents a clear strength, since most studies were carried out in both male and female populations encompassing a wide age range (21–80 years).

In conclusion, US features were less efficient in detecting malignancy in mixed nodules, which harbored malignant lesions as often as solid nodules in this population. Based on our findings, we believe that different criteria should be considered for US-guided FNAB in mixed nodules, and the recommended minimal size for US-guided FNAB should be unified for mixed and solid nodules.

## Figures and Tables

**Table 1:** Ultrasonographic features evaluated in each thyroid nodule.

Echostructure	Solid	Mixed	Cystic	Spongiform
Echogenic pattern	Hypoechoic	Hyperechoic	Isoechoic	Anechoic
Margins	Regular	Irregular		
Halo	Presence	Absence		
Microcalcifications	Presence	Absence		
Diameters (mm)	Longitudinal	Anteroposterior (AP)	Axial	

**Table 2:** General characteristics of mixed and solid nodules.

	Mixed nodules (*n* = 158)	Solid nodules (*n* = 304)	*P* value
Patients' age (years)	69 ± 7	69 ± 7	0.504
Longitudinal diameter (mm, *n* = 158/304)	21 (15–30)	17 (14–24)	<0.001
Anteroposterior diameter (mm, *n* = 147/281)	14 (10–20)	11 (9–16)	<0.001
Axial diameter (mm, *n* = 143/252)	16 (12–23)	13 (10–19)	<0.001
Hypoechoicity (%, *n* = 148/298)	53	54	0.950
Irregular margins (%, *n* = 129/258)	40	37	0.604
Microcalcifications (%, *n* = 133/269)	42	39	0.554
Absence of halo (%, *n* = 102/214)	54	58	0.451
Taller than wider (%, *n* = 140/252)	29	39	0.057
Malignant cytology (%, *n* = 136/256)	7.4	5.8	0.565

The values in brackets indicate the number of patients with available information for that specific variable. For continuous variables, results are expressed as mean ± SD and median (interquartile range) corresponding to Gaussian and non-Gaussian distribution, respectively.

**Table 3:** Associations between US features with malignant cytology in mixed and solid nodules.

Echo structure	US feature	Prevalence Benign (%)	Prevalence Malignant (%)	*P value	Sensitivity	Specificity	LR (CI95)	Posttest probability (%)
Mixed nodules (*n* = 135)	Hypoechoic (*n* = 125; *M*: 9)	50	56	0.767	0.56	0.50	1.10 (0.60–2.03)	NI
	Irregular margins (*n* = 111; *M*: 6)	36	33	0.887	0.33	0.64	0.92 (0.29–2.94)	NI
	Microcalcifications (*n* = 113; *M*: 8)	42	50	0.655	0.50	0.58	1.2 (0.57–2.47)	NI
	Absence of halo (*n* = 86; *M*: 6)	49	83	0.102	0.83	0.51	1.71 (1.12–2.61)	9.8
	Taller than wider (*n* = 135; *M*: 6)	26	50	0.209	0.50	0.74	1.88 (0.80–4.44)	10.7

Solid nodules (*n* = 256)	Hypoechoic (*n* = 250; *M*: 15)	53	50	0.805	0.50	0.47	0.94 (0.88–0.97)	NI
	Irregular margins (*n* = 218; *M*: 11)	36	73	0.015	0.73	0.63	2.00 (1.37–2.93)	11.3
	Microcalcifications (*n* = 225; *M*: 13)	39	69	0.029	0.69	0.61	1.79 (1.20–2.67)	10.3
	Absence of halo (*n* = 181; *M*: 9)	60	55	0.769	0.55	0.40	0.92 (0.51–1.67)	NI
	Taller than wider (*n* = 209; *M*: 8)	40	62	0.211	0.62	0.60	1.55 (0.88–2.72)	9.0

Indeterminate cytology nodules were discarded prior to the analysis. The values in brackets indicate the number of patients with available information for that specific variable and the total cases of malignant cytology. Pretest probability in this population was 0.06. NI: not improved compared to pretest probability. *P value corresponds to that obtained in the Chi-square test.

**Table 4:** Association of the combination between the presence of microcalcifications and/or irregular margins and the presence of malignant cytology.

Echostructure	Model	Wald	OR	95% CI	*P* value
Mixed (*n* = 118; *M*: 9)	Age	0.512	0.967	0.881–1.061	0.474
	Irregular margins-microcalcifications	0.077	1.128	0.484–2.626	0.781

Solid (*n* = 207; *M*: 15)	Age	1.710	0.940	0.857–1.031	0.191
	Irregular margins-microcalcifications	6.275	2.759	1.247–6.103	0.012

The values in brackets indicate the number of patients with available information for that specific variable and the total cases of malignant cytology included.
